# Effectiveness of intraoperative radiotherapy vs hypofractionated postmastectomy radiotherapy for early stage breast cancer: A protocol for systematic review and meta-analysis

**DOI:** 10.1097/MD.0000000000024098

**Published:** 2021-01-15

**Authors:** Jiang-Yi Feng, Ge Li, Yi Guo, Yun-Han Gao, Sha-Ying Ma

**Affiliations:** General Surgery Department, Chongqing University Central Hospital, Chongqing, China.

**Keywords:** breast cancer, hypofractionated postmastectomy radiotherapy, intraoperative radiotherapy, meta-analysis, protocol, systematic review

## Abstract

**Background:**

Radiotherapy is one of the essential components of breast cancer treatment. It destroys the remaining cells in the chest area after breast cancer surgery and is useful for reducing the necessity of mastectomies. As a single dose of radiation at the time of breast conserving surgery, intraoperative radiotherapy delivers radiotherapy directly and accurately to the tumor itself or the tumor bed whilst delivering minimal dose to the surrounding normal tissues. Hypofractionated postmastectomy radiotherapy with shorter and more convenient hypofractionated dose schedules might help to treat more patients and reduce cost. We will conduct a comprehensive systematic review and meta-analysis to compare the effectiveness of these 2 therapies in the management of early stage breast cancer.

**Methods:**

Four English databases (PubMed, Embase, Cochrane Library, and Web of Science) and 3 Chinese databases (China National Knowledge Infrastructure, China Science and Technology Journal Database, and Chinese Biomedical Literature Database) will be searched from inception of databases to December 2020 without language limitation. Two reviewers will independently conduct selection of studies, data extraction and management, and assessment of risk of bias. Any disagreement will be resolved by the third reviewer. Review Manager 5.3 (The Cochrane Collaboration, Software Update, Oxford, UK) will be used for data synthesis. Cochrane risk of bias assessment tool will be used to assess the risk of bias.

**Results:**

This study will provide a systematic synthesis of current published data to compare the effectiveness of intraoperative radiotherapy vs hypofractionated postmastectomy radiotherapy for early stage breast cancer.

**Conclusions:**

This systematic review and meta-analysis will provide clinical evidence for the effectiveness of intraoperative radiotherapy vs hypofractionated postmastectomy radiotherapy for early stage breast cancer, and inform our understanding of the value of intraoperative radiotherapy and hypofractionated postmastectomy radiotherapy for early stage breast cancer.

**Study registration number:**

INPLASY2020110115.

## 1 Introduction

Breast cancer has been the most common cancer and the primary cause of mortality due to cancer in women.^[1–3]^ In developed countries, the 5-year survival rate is about 80%, and in developing countries, it is about 40%.^[4]^ Enhancement in screening mammography and management is beneficial for detecting early stage breast cancer and reducing mortality due to breast cancer.^[5]^ Surgery such as lumpectomy, mastectomy and reconstructive surgery is the foremost management strategy for breast cancer patients.^[6,7]^ Radiotherapy is one of the essential components of breast cancer treatment. It is useful for reducing the necessity of mastectomies and destroys the remaining cells in the chest area after breast cancer surgery.^[8–10]^ Many randomized controlled trials (RCTs) and meta-analyses reported that radiotherapy as an adjuvant treatment showed benefits in both locoregional control and overall survival.^[11–15]^

Intraoperative radiotherapy is a single dose of radiation at the time of breast conserving surgery. It has been used in a range of tumor sites, including the breast, head and neck, lung, and so on.^[16–18]^ Intraoperative radiotherapy delivers radiotherapy directly and accurately to the tumor itself or the tumor bed whilst delivering minimal dose to the surrounding normal tissues.^[19]^ One study showed that the recurrence rate of ipsilateral breast tumor in intraoperative radiotherapy group was significantly greater than in external radiotherapy group, and overall survival did not differ between groups.^[20]^

Hypofractionated postmastectomy radiotherapy with shorter and more convenient hypofractionated dose schedules might help to treat more patients and reduce cost.^[21]^ One study showed hypofractionated postmastectomy radiotherapy was non-inferior to and had similar toxicities to conventional fractionated radiotherapy in high-risk breast cancer patients, and it provided better convenience.^[22]^ One study found that Hypofractionated postmastectomy radiotherapy is cost-effective compared with conventional radiotherapy and intraoperative radiotherapy for early stage breast cancer patients.^[23]^ Guenzi M et al evaluated the local recurrence in patients with early breast cancer who underwent intraoperative radiationtherapy or hypofractionated postmastectomy radiotherapy, and the result showed that at 6 years a significant higher rate of local recurrence occurred in intraoperative radiationtherapy group.^[24]^

Up to now, no systematic review or meta-analysis has been performed to compare the effectiveness of intraoperative radiotherapy vs hypofractionated postmastectomy radiotherapy for early stage breast cancer. Therefore, we will conduct a comprehensive systematic review and meta-analysis to compare the effectiveness of these 2 therapies in the management of early stage breast cancer.

## 2 Methods

### 2.1 Study registration

This study has been registered on INPLASY (INPLASY2020110115). This meta-analysis will be performed following Preferred Reporting Items for Systematic Reviews and Meta-Analyses (PRISMA) statement checklist.^[25]^

### 2.2 Eligibility criteria for study selection

#### 2.2.1 Types of studies

RCTs comparing the effectiveness of intraoperative radiotherapy vs hypofractionated postmastectomy radiotherapy for early stage breast cancer will be included without language limitation.

#### 2.2.2 Types of participants

Participants diagnosed with early stage breast cancer will be included.

#### 2.2.3 Types of interventions

In the treatment group, patients were given intraoperative radiotherapy. It the control group, patients were given hypofractionated postmastectomy radiotherapy.

#### 2.2.4 Types of outcomes

The recurrence rate and the rate of 5-year disease-free survival will be designated as the primary outcome. Adverse events will be designated as the secondary outcome.

### 2.3 Search strategy

Four English databases (PubMed, Embase, Cochrane Library, and Web of Science) and 3 Chinese databases (China National Knowledge Infrastructure, China Science and Technology Journal Database, and Chinese Biomedical Literature Database) will be searched from inception of databases to December 2020 without language limitation. Additional trials will be searched by reviewing the reference lists of the retrieved articles, conference proceedings, and gray literature. The detailed search strategy for PubMed is shown in Table 1. The similar search strategies will be used for other electronic databases.

**Table 1:** Search strategy of PubMed.

Number	Search terms
1	breast cancer
2	breast carcinoma
3	mammary cancer
4	breast tumor
5	Or 1–4
6	intraoperative radiotherapy
7	IORT
8	intra-operative radiation therapy
9	intraoperative irradiation
10	Or 6–9
11	hypofractionated
12	hypofractionated radiation therapy
13	hypofractionated radiotherapy
14	postmastectomy radiotherapy
15	Or 11–14
16	Randomized controlled trial
17	Clinical trial
18	Random
19	Trial
20	Or 16–19
21	5 and 10 and 15 and 20

### 2.4 Selection of studies

The searched articles will be integrated into Endnote X7 (Thomas Reuters, CA) and duplicates will be excluded by software. Two reviewers will independently scan titles and abstracts according to the eligibility criteria. The remaining records will be read by full texts. Then final included studies will be determined. Any disagreement will be resolved by the third reviewer. A PRISMA flowchart will be designed to describe the details of selection process.

### 2.5 Data extraction and management

Two reviewers will independently conduct data extraction. Any disagreement will be resolved by the third reviewer. The following information from each study will be extracted: authors name, publication year, journal, study design, participants baseline characteristics, number of participants in each arm, experimental intervention, control intervention, and outcomes. If the trials have more than 2 arms, only the interest-reported information and data will be extracted. We will try our best to contact original authors for detailed information by email or phone if required information is missing.

### 2.6 Assessment of risk of bias

Cochrane risk of bias assessment tool will be used to assess the risk of bias of the included studies. Seven items such as random sequence generation, allocation concealment, blinding of participants and personnel, blinding of outcome assessment, incomplete outcome data, selective reporting and other bias will be evaluated by 2 reviews independently. Any disagreement will be resolved by the third reviewer.

### 2.7 Data synthesis and analysis

#### 2.7.1 Data synthesis

Review Manager 5.3 (The Cochrane Collaboration, Software Update, Oxford, UK) will be used for data synthesis. Risk ratio will be used for dichotomous outcomes with 95% confidence interval. Continuous outcomes will be presented as mean difference or standardized mean difference with 95% confidence interval. We will use *I*^2^ test to identify heterogeneity. The *I*^2^ value >50% means significant heterogeneity, and the random effects model will be used. The *I*^2^ value ≤50% means minor heterogeneity, and the fixed effects model will be utilized.

#### 2.7.2 Subgroup analysis

Subgroup analysis will be performed based on the different participant characteristics and outcome indicators to check the potential heterogeneity and inconsistency. If significant heterogeneity still exists after subgroup analysis, meta-analysis will not be pooled, and descriptive summary will be reported.

#### 2.7.3 Sensitivity analysis

Sensitivity analysis will be performed to test the robustness and reliability of pooled results by eliminating studies in low quality.

#### 2.7.4 Reporting bias

Publication bias will be assessed with funnel plot and Egger regression analysis if sufficient trials (≥10 trials) are included.

#### 2.7.5 Ethics and dissemination

Ethical approval is not necessary because this study is based on literature analysis. The results of this study will be published in a peer-reviewed journal.

## 3 Discussion

To our knowledge, this is the first systematic review and meta-analysis to conduct a comprehensive literature search and provide a systematic synthesis of current published data to compare the effectiveness of intraoperative radiotherapy vs hypofractionated postmastectomy radiotherapy for early stage breast cancer. Four English databases and 3 Chinese databases will be searched to avoid missing any potential eligible studies, and apply rigorous methodology to examine studies reporting intraoperative radiotherapy vs hypofractionated postmastectomy radiotherapy for early stage breast cancer. This systematic review and meta-analysis will provide clinical evidence for the effectiveness of intraoperative radiotherapy vs hypofractionated postmastectomy radiotherapy for early stage breast cancer, and inform our understanding of the value of intraoperative radiotherapy and hypofractionated postmastectomy radiotherapy. We believe that the conclusions drawn from this study may be beneficial to patients, clinicians, and policy makers.

## Author contributions

**Conceptualization**: Jiang-yi Feng, Ge Li, Sha-ying Ma.

**Data curation**: Jiang-yi Feng, Ge Li, Yi Guo.

**Formal analysis**: Ge Li, Yi Guo, Yun-han Gao.

**Investigation:** Ge Li, Yi Guo, Yun-han Gao.

**Methodology**: Jiang-yi Feng, Ge Li, Sha-ying Ma.

**Project administration**: Sha-ying Ma.

**Resources**: Jiang-yi Feng, Ge Li.

**Software**: Yi Guo, Yun-han Gao.

**Supervision**: Yi Guo, Yun-han Gao.

**Validation**: Sha-ying Ma.

**Visualization**: Jiang-yi Feng.

**Writing – original draft**: Jiang-yi Feng, Ge Li.

**Writing – review & editing**: Jiang-yi Feng, Ge Li, Yi Guo, Yun-han Gao, Sha-ying Ma.

## Abbreviations

Abbreviations: PRISMA = Preferred Reporting Items for Systematic Reviews and Meta-Analyses, RCTs = randomized controlled trials.
